# SG-APSIC1136: Multidrug-resistant organisms: Elevating issues identified by antimicrobial stewardship to improve infection control responses

**DOI:** 10.1017/ash.2023.77

**Published:** 2023-03-16

**Authors:** Frances Sheehan, Meru Sheel, Karina Kennedy, Kathryn Daveson

**Affiliations:** Australian National University, Canberra, Australia; Australian National University, Canberra, Australia; The Canberra Hospital/Australian National University, Canberra, Australia; The Canberra Hospital, Canberra, Australia

## Abstract

**Objectives:** Resistance to third-generation cephalosporins in *Escherichia coli* bacteremia is on the rise in Australia. Currently, laboratory definitions of multidrug-resistant organisms determine infection control responses. The incidence of particular extended-spectrum β-lactamase (ESBL) *E. coli* phenotype, with nonsusceptibility to both ciprofloxacin and trimethoprim-sulfamethoxazole, is increasing in the Australian Capital Territory, Australia. The increase was noted primarily through antimicrobial stewardship clinical care rather than standard infection control or microbiology processes. Clinically, patients are left with limited or no oral therapeutic options for treatment. Despite not necessarily meeting the laboratory definition of a multidrug-resistant organism, this phenotype is likely to be just as transmissible as other healthcare-associated pathogens. We sought to determine whether laboratory definitions of multidrug-resistant organisms adequately inform infection control responses. **Methods:** Using laboratory data from Australian Capital Territory (ACT) Pathology, we identified all ESBL *E. coli* bloodstream isolate episodes from 2016 to 2020. We then reviewed the antibiotic sensitivities of each isolate to identify isolates with nonsusceptibility to both ciprofloxacin and trimethoprim-sulfamethoxazole. We then compared these isolates with the multidrug-resistant organism definition used by ACT Pathology. **Results:** In total, 152 isolates were reviewed. ACT Pathology classified 35 (23.0%) of these isolates as a multidrug-resistant organisms. We identified 80 (52.6%) isolates with nonsusceptibility to both ciprofloxacin and trimethoprim-sulfamethoxazole. Of these 80 isolates, only 24 (30.0%) met the ACT Pathology definition of a multidrug-resistant organism. **Conclusions:** Multidrug-resistant organism definitions should encompass a broad range of healthcare considerations. When the laboratory defines what is important, it may not include the complete spectrum of clinical care concerns. To help combat the rise of multidrug-resistant organisms, definitions for organisms of resistance and transmissibility significance should be developed in conjunction with microbiology, infection control, and antimicrobial stewardship.

